# Effect Of A “No Superuser Opioid Prescription” Policy On ED Visits And Statewide Opioid Prescription

**DOI:** 10.5811/westjem.2017.6.33414

**Published:** 2017-07-25

**Authors:** Zachary P. Kahler, Paul I. Musey, Jason T. Schaffer, Annelyssa N. Johnson, Christian C. Strachan, Charles M. Shufflebarger

**Affiliations:** * Indiana University School of Medicine, Department of Emergency Medicine, Indianapolis, Indiana; † Indiana University Health Methodist Hospital, Indianapolis, Indiana; ‡ University of South Carolina, Greenville School of Medicine, Department of Emergency Medicine, Greenville, South Carolina

## Abstract

**Introduction:**

The U.S. opioid epidemic has highlighted the need to identify patients at risk of opioid abuse and overdose. We initiated a novel emergency department- (ED) based interventional protocol to transition our superuser patients from the ED to an outpatient chronic pain program. The objective was to evaluate the protocol’s effect on superusers’ annual ED visits. Secondary outcomes included a quantitative evaluation of statewide opioid prescriptions for these patients, unique prescribers of controlled substances, and ancillary testing.

**Methods:**

Patients were referred to the program with the following inclusion criteria: ≥ 6 visits per year to the ED; at least one visit identified by the attending physician as primarily driven by opioid-seeking behavior; and a review by a committee comprising ED administration and case management. Patients were referred to a pain management clinic and informed that they would no longer receive opioid prescriptions from visits to the ED for chronic pain complaints. Electronic medical record (EMR) alerts notified ED providers of the patient’s referral at subsequent visits. We analyzed one year of data pre- and post-referral.

**Results:**

A total of 243 patients had one year of data post-referral for analysis. Median annual ED visits decreased from 14 to 4 (58% decrease, 95% CI [50 to 66]). We also found statistically significant decreases for these patients’ state prescription drug monitoring program (PDMP) opioid prescriptions (21 to 13), total unique controlled-substance prescribers (11 to 7), computed tomography imaging (2 to 0), radiographs (5 to 1), electrocardiograms (12 to 4), and labs run (47 to 13).

**Conclusion:**

This program and the EMR-based alerts were successful at decreasing local ED visits, annual opioid prescriptions, and hospital resource allocation for this population of patients. There is no evidence that these patients diverted their visits to neighboring EDs after being informed that they would not receive opioids at this hospital, as opioid prescriptions obtained by these patients decreased on a statewide level. This implies that individual ED protocols can have significant impact on the behavior of patients.

## INTRODUCTION

### Background

In the early 1990s there was a concerted effort by the Veterans Health Administration (VHA) and The Joint Commission to target pain management with opioids.^1^ Pain quickly became the “fifth vital sign,” and opioid prescriptions escalated.^2^ Between 1999 and 2010, the marketing of opioids to pharmacists, hospitals, and doctors’ offices had quadrupled, and there was a 300% increase in the prescription of opioids in the U.S.^3^,^4^ With this dramatic increase in opioid prescribing behavior, a number of serious unintended consequences were noted. In 2008, prescription opioids were estimated to be the direct cause for approximately 15,000 annual overdose deaths in the U.S., with that number almost doubling to 29,000 in 2014. 5,6 Each opioid abuser incurs $20,546 more in annual healthcare costs than demographically similar controls.^7^ Direct healthcare costs of improper and non-medical opioid prescription use is estimated to be greater than $72 billion per year.^3^

### Importance

Emergency department (ED) visits related to prescription opioid abuse have risen dramatically from 173,000 in 2004 to 416,000 in 2009 and now are over 500,000 annually.^3^,^4^,^7^ Many efforts have been made to identify patients with drug-seeking behaviors as well as providers with aberrant prescribing practices.^7^–^11^ These include increased regulations on pain clinics, prescription threshold guidelines, controlled substance contracts, and the establishment of prescription drug monitoring programs (PDMP).

### Goals of This Investigation

The purpose of this study was to examine and present the outcomes of a novel interventional chronic pain program established in a metropolitan ED. The protocol was designed to transition superuser opioid-seeking patients out of the ED and into a chronic taper-to-abstinence pain program. We primarily hypothesized that visits to the ED post-referral would decrease. We hypothesized that secondary outcomes would similarly decrease, such as statewide opioid prescriptions, number of opioid prescribers, number of electrocardiographs (ECG), laboratory tests, radiographs, and computed tomography (CT) imaging.

## METHODS

### Study Design and Setting

This study is a retrospective analysis of a novel preexisting, administrative chronic pain management program at Methodist Hospital in Indianapolis, IN. This is an urban teaching hospital with an annual ED volume of approximately 102,000 patients per year. Patients were drawn from the existing administrative database of frequent opioid recidivists who had been prospectively identified for inclusion into the program as outlined below. The study is designed as a one-way crossover intervention, with patients serving as their own controls in the year prior to their referral in the program. The protocol was approved as an administrative policy four years prior to the collection of any research data. Research data gathering was separately approved and registered by the Indiana University (IU) Institutional Review Board (1409177708).

Population Health Research Capsule

What do we already know about this issue?

Opioid prescriptions and overdoses have increased significantly in the past 30 years. Superuser patients may use the Emergency Department (ED) as a source for opioids.

What was the research question?

Does ED referral to a pain management group – with subsequent EMR-based reminders to ED practitioners - decrease annual visits from superuser patients?

What was the major finding of the study?

Superuser patients had fewer overall ED visits after the intervention, decreasing annual visits from 14 to 4.

How does this improve population health?

Enrollment in a chronic pain program with EMR-based provider reminders appeared to decrease overall visits to the ED post-intervention.

### Selection of Participants

Inclusion criteria into the chronic pain program were as follows: 1) Frequent use of the ED, defined as ≥ 6 visits per year; 2) At least one visit identified by the treating attending physician as primarily driven by opioid-seeking behavior; and 3) Chart review by ED administration and case management for evidence of ED misuse. Patients meeting all three of these criteria were referred to the chronic pain program unless they met exclusion criteria below.

Exclusion criteria for the chronic pain program were preexisting chronic disease processes expected to cause frequent and uncontrollable visits to the ED, such as cancer or sickle cell disease. Pregnancy and age were not exclusion criteria.

We excluded patients from the retrospective data analysis if they had not been part of the chronic pain program for at least a year. These patients would not have a full year of data post-intervention to compare to the year prior. Demographic characteristics of the participants can be found in Table 1.

### Interventions

After medical director approval, patients were referred to a free, outpatient taper-to-abstinence pain management clinic. A chronic pain management and addiction specialist runs the clinic. Patients were notified by an administrator either in person at their next visit or by telephone that they had been referred into a chronic pain program. They were also informed that they would no longer routinely receive opioids or opioid prescriptions for their chronic painful conditions from the ED. Additionally, they received written instructions and information either in person or by certified mail.

Exceptions were made for acute pain not related to a chronic condition, such as new fractures. Those patients non-compliant with follow-up with the pain management program were contacted on subsequent ED visits and referred again. Treatment with opioids, both parenteral and prescribed, remained at the discretion of the treating ED provider, with a reminder in place that the patient had already been given outpatient follow-up.

To reinforce the program to emergency physicians, an electronic medical record- (EMR) based notification was implemented, which was activated any time the patient arrived in the ED. This notification is three-fold. On the ED tracking screen, a flag is placed to alert providers to the patient’s referral to the program. Upon opening the patient’s chart, a pop-up alert indicates the patient’s chronic pain management, with instructions to refer to the case management notes for specific details. If opioids are chosen as a treatment modality, a separate notification activates to ensure that the provider is aware that the patient has been referred to pain management.

### Methods and Measurements

We collected and managed study data using REDCap electronic data capture tools hosted at IU.^12^ REDCap (Research Electronic Data Capture) is a secure, web-based application designed to support data capture for research studies, providing the following: 1) an intuitive interface for validated data entry; 2) audit trails for tracking data manipulation and export procedures; 3) automated export procedures for seamless data downloads to common statistical packages; and 4) procedures for importing data from external sources.

Data regarding controlled substance prescriptions pre- and post-referral into the program was captured via the Indiana Board of Pharmacy Prescription Monitoring System or INspect. All pharmacies are mandated to report any controlled substance filled to this database. The few exceptions are entities and clinics governed by federal regulations (the VHA, as well as methadone and suboxone clinics). Data included in the database are the following: Patient demographics (name, date of birth, address), prescriber, prescriber demographics, date of prescription filled, pharmacy demographics, prescribed substance, strength, quantity, intended days, and date written. We obtained data regarding ED visits pre- and post-referral via Indiana University Health’s (IUH) EMR (Cerner), which connects approximately 32 hospitals, rehabilitation facilities, and clinics across the greater Indianapolis area. Data were collected on a data abstraction form by blinded, trained abstractors. As this was a retrospective chart review, a random sample of 10% of the charts was reassessed by one of the investigators (reassessed by one of the investigators [ZPK]). We performed a kappa analysis for the primary outcome, which was found to be 0.91, indicating excellent inter-rater reliability.

### Outcomes

The primary outcome of interest was the difference between annual ED visits to an annual ED visits to an IUH ED pre- and post-referral into the chronic pain program. Secondary outcomes included their statewide opioid prescriptions, number of unique prescribers of controlled substances, as well as ancillary testing: number of ECGs, laboratory tests, radiographs, and CTs.

### Analysis

We performed statistical analysis using the R statistical software package with the Rstudio frontend (Foundation for Open Access Statistics, Boston MA). Descriptive analyses are reported where appropriate. Mean values are reported for normal data with standard deviations, with significance between results analyzed with Student’s t-test for unpaired values and Student’s paired t-test for paired values. Non-normal data is reported in medians with interquartile ranges (IQR), with significance analyzed by Mann-Whitney U test for unpaired values and Wilcoxon signed-rank test for paired values. As most of the data is non-normal, the primary analysis method used was the Wilcoxon signed-rank test for paired medians. We calculated data to a significance of α=0.05 and β=0.20 where appropriate. There was no formal sample size calculated, as this was a retrospective study performed on all eligible existing patients in the program. A priori to capture and analysis of our data, we identified annual ED visits as our primary outcome, with secondary outcomes as described in outcomes and analysis.

## RESULTS

### Characteristics of Study Subjects

At the time of data gathering, 278 patients had been referred into the program. Of those patients, 243 had been in the program for one year or greater, and therefore had 12 months of data both pre- and post-intervention. Demographics of the participants are shown in Table 1. Mean age of the study group was 41, 62% of which were female. These were predominantly White patients (70%), while 29% were Black, and the remainder American Indian, Asian, and Hispanic. The cohort was healthy in general with a median Charleson Comorbidity score of 1. The most common comorbidities in this cohort were chronic pulmonary disease (asthma or COPD) n=23, and diabetes without end-organ dysfunction n=23.

The primary outcome was the number of ED visits pre- and post-intervention. This data is represented visually in Figure.

Given the skewness of the data, our data is primarily reported in median values, although mean values are also reported in Table 2.

Median ED visits to hospitals in our health system decreased from 14 to 4 (58% decrease, 95% CI [50 to 66]). We evaluated visits as paired data, with each patient serving as their own control. Mean visits decreased from 19 to 6, implying a rightward skewness of the data. When assessing the highest quartile, we found that median visits decreased from 25 to eight. The outlier patient decreased annual visits to our ED from 131 to 13.

Secondary outcomes were similarly significant. Total median number of opioid prescriptions filled statewide decreased from 21 to 13 (30% decrease, 95% CI [24 to 37]), as did median number of statewide prescribers 11 to seven (31% decrease, 95% CI [23 to 38]).

## DISCUSSION

In this study, we present the outcomes of a novel, administratively instituted “no-opioid” policy for 243 patients at a large metropolitan hospital. These patients had been identified as over-using the ED, primarily to obtain opioids for chronic pain. It has been previously estimated that approximately 5% of patients account for 25% of all ED visits, and chronic pain and addiction is often a driving force behind this recidivism.^13^ This population is at high risk for opioid overdose and subsequent hospitalization, and a major component of their access to opioids is a “revolving door” of prescribers.^14^

Our study demonstrated decreased visits to our facility from these patients by 58%, a decrease in the number of unique prescribers for their controlled substances by 31%, and a decrease in the number of prescriptions these patients received statewide by 30%. Of note, our intervention appears to have decreased overall opioid prescriptions and prescribers statewide for these enrolled patients, despite being implemented at only one facility*.* We believe that this implies that there is a significant degree of local bias in care for these patients – that is, patients preferentially seek care at the closest ED to their home. When access to opioids is fettered at that site, these patients did not appear to supplement by simply visiting neighboring EDs. This may be the understated strength of our administrative policy; our results imply that our ED was a major source of these patients’ legal access to opioids. Further, when opioid prescriptions are restricted from the ED, this patient population decreases ED visit frequency.

To date, there is no standardized definition of frequent users of the ED. Various authors have proposed anywhere from 3–10 visits as “frequent.”^15^–^18^ Our protocol used ≥ 6 visits per year as the cutoff, although the median number of visits in our study population overall was 14 per year, with the highest 10% of our study group visiting 52 times per year. This population can be very difficult to manage; psychosocial factors, addiction, opioid hyperalgesia, and personality traits influence their presentation. Further, emergency physicians often treat acute flares of chronic pain with a “short course” of opioids, which may reinforce the patient’s ED recidivism.

Several researchers have evaluated ED pain protocols prior to this study.^17^–^19^ Our study is unique for three main reasons: the large number of patients included, the analysis of repeat ED visits, and the EMR-based reminders to providers. To date, no study has evaluated a policy such as ours on such a scale, nor have studies evaluated the granular effect on resource expenditure. We believe the success of this protocol was wholly dependent on strong administrative support for the policy and the repeated EMR-based alerts.

With regards to the first point, emergency providers often feel obligated to acquiesce to patient demands for fear of lowered patient satisfaction scores, or simply to avoid a complaint.^2^,^20^ However, recent studies have called into question this assumption, with at least one study by Schwartz et al. finding no association between opioid administration and patient satisfaction. 2,21–24 Germaine to this point, recent evidence has demonstrated that increasing patient satisfaction scores are correlated with increased prescription drug expenses, increased healthcare expenditures and increased mortality.^25^ It is of paramount importance to provide medically appropriate care for patients, which may contrast with the goals of ED superusers. This subset of patients often requests unnecessary treatments, inappropriate prescriptions, or may simply use the ED as a food and bed source. Thus, we feel that strong administrative support for a policy such as ours is critical.

As only a handful of patients followed up with the pain management clinic, this component of the intervention is unlikely to have changed ED visit frequency or prescription volume. Instead, the authors believe that the EMR-based reminders to physicians were the key component. These reminders occurred at every visit for each provider who interacted with the patient. Thus, prior to the administration or prescription of an opioid, the EMR reinforced the behavior of physicians and therefore patients. Many hospitals use a provider-initiated system for opioid management; that is, a provider must go looking for a care management note, chronic pain policy, or must do a chart review manually.^19^ While accessing the state PDMP is encouraged before prescription of controlled substances, this typically requires a provider to leave the EMR and log into a separate system, which may hinder its use.

Our EMR-based alerts were active, informing the patient’s ED provider of their chronic pain referral at every visit. When a patient’s chart is initially accessed at each visit, a reminder screen is displayed prior to chart access and order entry. This ensured that every provider who interacted with the patient was aware of the referral to the clinic. A secondary alert was triggered if providers ordered opioids.

Ancillary testing decreased proportionally to the decrease in ED visits. If our intervention had biased physicians against these patients, causing physicians to assume that they were never “sick,” one would expect a comparatively greater decrease in the quantity of ancillary services used. Instead, there was strong correlation between both the decrease in ED visits and most of the secondary outcome variables, implying that these patients received the same amount of testing at each visit. To a degree, this is surprising; if a patient routinely presents with opioid-seeking behavior, one would plausibly expect providers to decrease testing.

Of all 278 patients referred into the program, to date only seven have followed up with the referral outpatient clinic. Of these, only three have continued to follow up with the clinic, and the other four were discharged for noncompliance with controlled substance covenants. The remaining 271 patients either already had a primary care physician or missed all scheduled appointments with the clinic. We posit that this extremely poor compliance is a direct function of the patient population included in our protocol; prior to referral in the protocol these patients had a 14:1 median ratio of ED visits to clinic visits. Our chronic pain clinic ensured that these patients had guaranteed follow-up for their chronic pain, and thus the ED was no longer an acceptable mechanism to fill this need.

There has been much research into maintenance therapy, adjunct therapy, and replacement therapy for opioid cessation.^26^–^33^ Unfortunately these clinics and therapies are often expensive, and due to the nature of the patients enrolled in this study, many had no resources or had already violated a pain contract. Thus, we identified a provider who was willing to see all of these patients free of charge, provided they were willing to wean to abstinence. Recent research has identified success with ED-initiated buprenorphine treatment as compared to intervention or referral to community resources.^27^ Our intervention was initiated without financial support; if possible, we would recommend that significant support in the form of social work, case management, and addiction specialists be provided to this vulnerable population.

One of our main concerns is that when cut off from a source of opioids, these patients may resort to illicit methods to supply their addiction. Recently there was an HIV outbreak in Southern Indiana among Opana (oxymorphone) users who were sharing needles to inject their prescriptions.^34^ While the risk of illicitly obtaining opioids is a major public health concern, the authors do not feel that this is an appropriate rationale for the ED to provide unfettered access to these medicines. Instead, we believe that the revolving door of the ED contributes to the problem. Future work is planned to aggressively support these patients with addiction management, social work, case management, and replacement therapy.

## LIMITATIONS

There are several limitations to this study. This was a retrospective observational analysis of a preexisting administrative database at a large metropolitan hospital, which is part of a wide-reaching health system. While the data itself were not prospectively collected, the administrative protocol was performed in a prospective fashion. Thus, this study was a retrospective analysis of a prospective protocol, which we feel improved the robustness of the data. However, fundamentally this study was a retrospective review of existing data, and limitations exist for this form of study protocol. There is a potential for sampling bias in any retrospective review, although our abstractors followed strict rules and were transcribing concrete data points. We do note that we were unable to blind abstractors, but given the cohort nature of the study, all patients were “case” and blinding would be impossible.

While the EMR connects most of the hospitals in our healthcare system, we were unable to assess visits at most urgent visit centers, or EDs within other health systems. However, the use of our state PDMP does act as a surrogate for whether subjects simply shifted their ED use to other health systems. We were unable to determine total morphine equivalents for our patients. Thus, the decrease in annual prescriptions may in fact represent consolidation of prescriptions to fewer providers, with increased pill quantities per prescription. As there was no interview component to this study, we cannot determine if these patients went on to use illicit drugs at an increased rate.

One potential confounder is the increased national attention on restriction of opioid prescription at the same time as the study period. However, the study authors feel that it is unlikely that national attention alone resulted in a 58% decrease in opioid prescriptions for these patients.

A second confounder may be a natural decrease in opioid usage as painful conditions improve. However, prior studies have demonstrated that patients on long-term opioid therapy are unlikely to discontinue usage.^35^,^36^ Thus, we conclude that our intervention was the cause for the decreased overall prescription rate.

## CONCLUSION

In summary, this study demonstrates the efficacy of an interventional protocol intended to decrease ED visits among ED superusers. These patients were selected as those who frequently presented for the primary purpose of obtaining opioids for chronic pain. While only a handful took advantage of the chronic pain clinic to which they were referred, our protocol resulted in a decrease in ED visits, fewer statewide opioid prescriptions for the cohort, and less ancillary testing such as ECGs, CTs and radiographs. Implementing this protocol is fairly straightforward, requiring only an EMR flag and a task force willing to steward the database. We believe that this protocol streamlines patient care, decreases unnecessary visits to the ED, improves patient safety and can be one tool to help EDs combat our current opioid epidemic.

## Figures and Tables

**Figure:**
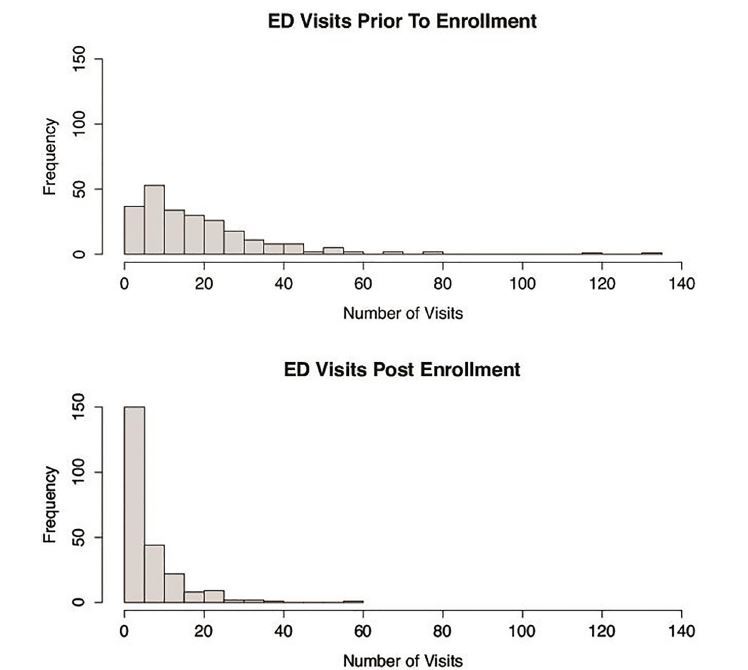
Pre- and post-intervention frequency (number of patients) of annual visits by patients identified as over-users of the emergency department (ED).

**Table 1:** Demographics with descriptive statistics.

Age (yrs)	
Mean (range)	41 (18–67)
Gender	
Female	151 (62%)
Race/ethnicity^*^	
American Indian	1 (0.4%)
Asian	1 (0.4%)
Hawaiian/Pacific Islander	0 (0%)
Black	71 (29%)
White	169 (70%)
Other	0 (0%)
Hispanic	3 (1%)
Charleson comorbidity score	
Median (IQR)	1 (0–1)

**Table 2:** Pre-post results for patients in the chronic pain program at one year.

	Median (IQR)	Mean	Min^ – Max	Percentage decrease (95% CI)
Number of ED visits				
Pre	14 (8 – 25)	19	0 – 131	58 (50 to 66)
Post	4 (2 – 8)	6	0 – 58	
Number of opioid prescriptions				
Pre	21 (12 – 30)	23	0 – 105	30 (24 to 37)
Post	13 (5 – 24)	16	0 – 103	
Number of prescribers				
Pre	11 (7 – 16)	13	0 – 45	31 (23 to 38)
Post	7 (3 – 12)	9	0 – 43	
Number of pharmacies used				
Pre	6 (3 – 9)	7	0 – 34	29 (14 to 36)
Post	5 (2 – 7)	5	0 – 17	
Number of lab draws				
Pre	47 (17 – 101)	59	0 – 175	46 (36 to 57)
Post	13 (4 – 40)	31	0 – 184	
Number of radiographs				
Pre	5 (2 – 10)	8	0 – 64	44 (38 to 56)
Post	1 (0 – 5)	3	0 – 28	
Number of CTs				
Pre	2 (1 – 5)	4	0 – 32	63 (50 to 75)
Post	0 (0 – 2)	1	0 – 16	
Number of ECGs				
Pre	4 (1 – 12)	12	0 – 158	50 (38 to 67)
Post	2 (0 – 4)	4	0 – 49	
Number of clinic visits				
Pre	1 (0 – 5)	4	0 – 30	13 (−13 to 38)
Post	1 (0 – 4)	3	0 – 37	
Number of hospitalizations				
Pre	0 (0 – 1)	1	0 – 16	*
Post	0 (0 – 1)	0	0 – 7	
Number of hospital days				
Pre	0 (0 – 5)	7	0 – 207	85 (57 to 129)
Post	0 (0 – 0)	2	0 – 75	

*CT*, computerized tomography; *ED*, emergency department; *ECG*, electrodiogram; *IQR*, interquartile range.

Rank test for median value.

^ No minimum number of prescriptions required for referral. 8 patients unable to be found in EMR.

* Unable to compute secondary to divide by zero errors.
